# Fabrication of a Nickel Ferrite/Nanocellulose-Based Nanocomposite as an Active Sensing Material for the Detection of Chlorine Gas

**DOI:** 10.3390/polym14091906

**Published:** 2022-05-06

**Authors:** Nurjahirah Janudin, Noor Azilah Mohd Kasim, Victor Feizal Knight, Mohd Nor Faiz Norrrahim, Mas Amira Idayu Abdul Razak, Norhana Abdul Halim, Siti Aminah Mohd Noor, Keat Khim Ong, Mohd Hanif Yaacob, Muhammad Zamharir Ahmad, Wan Md Zin Wan Yunus

**Affiliations:** 1 Research Centre for Chemical Defence, Universiti Pertahanan Nasional Malaysia, Kem Perdana Sungai Besi, Kuala Lumpur 57000, Malaysia; faiznorrrahim@gmail.com (M.N.F.N.); masamira920724@gmail.com (M.A.I.A.R.); norhana@upnm.edu.my (N.A.H.); s.aminah@upnm.edu.my (S.A.M.N.); ongkhim@upnm.edu.my (K.K.O.); 2 Centre for Defence Foundation Studies, Department of Chemistry and Biology, Universiti Pertahanan Nasional Malaysia, Kem Perdana Sungai Besi, Kuala Lumpur 57000, Malaysia; 3 Wireless and Photonic Research Centre (WiPNET), Faculty of Engineering, Universiti Putra Malaysia, Serdang 43400, Malaysia; hanif@upm.edu.my; 4 Malaysia Agricultural Research and Development Institute, Lot PT3747, Jalan Tambun Tulang, Arau 02600, Malaysia; zamharir@mardi.gov.my; 5 Centre for Tropicalisation, Universiti Pertahanan Nasional Malaysia, Kem Perdana Sungai Besi, Kuala Lumpur 57000, Malaysia; wanmdzin@upnm.edu.my

**Keywords:** nickel ferrite, nanocellulose, chlorine gas, magnetic material, sensor

## Abstract

Chlorine gas is extensively utilised in industries as both a disinfectant and for wastewater treatment. It has a pungent and irritating odour that is comparable with that of bleach and can cause serious health issues such as headaches and breathing difficulties. Hence, efficiently, and accurately monitoring chlorine gas is critical to ensure that no undesirable incidents occur. Due to its remarkable characteristics, numerous researchers have explored the potential of ferrite nanoparticles as a sensing material for chlorine gas detection. Among several ferrite nanoparticles, nickel ferrite (NiFe_2_O_4_) is extensively studied as an inverse spinel structured magnetic material that may be ideal for sensing applications. However, the magnetic characteristics of NiFe_2_O_4_ cause agglomeration, which necessitates the use of a substrate for stabilisation. Therefore, nanocellulose (NC), as a green and eco-friendly substrate, is ideal for stabilising bare nickel ferrite nanoparticles. In a novel experiment, nickel ferrite was loaded onto NC as a substrate using in situ deposition. The structure was confirmed by X-ray Diffraction (XRD) analysis, while elemental composition was verified by Energy dispersive X-ray (EDX) analysis. Gas sensing properties were determined by evaluating sensitivity as a function of various regulating factors, such as the amount of nickel ferrite, gas concentration, repeatability, and reusability. In the evaluation, 0.3 g nickel ferrite showed superior response and sensitivity than those of other samples. The achieved response time was around 40 s, while recovery time was about 50 s. This study demonstrates the potential of a nickel ferrite/nanocellulose-based nanocomposite to efficiently monitor chlorine gas.

## 1. Introduction

Gases such as ammonia, benzene, carbon dioxide, hydrogen sulphide, carbon monoxide, and chlorine are readily available and can cause serious health concerns when they are present in the environment without being safely contained [1,2,3,4,5]. Chlorine is widely used in a variety of applications in both household and industrial environments, including its use as a disinfecting agent in swimming pools and drinking water to kill harmful pathogens [6], as bleach in the manufacture of fabrics, paper products, insecticides, rubber products, and solvents [7,8,9,10,11,12]. In fact, when chlorinated bleach is combined with certain cleaning products, it can form chlorine gas. Furthermore, during World War I, chlorine gained notoriety when it was utilised as a chemical warfare agent (CWA), and it has since been classified as a CWA choking agent. Chlorine gas is yellowish green and is often delivered under pressure and kept in liquid form. It is recognised by its strong and irritating odour, which provides early warning to those who become exposed to it. While it is not flammable, it may react explosively with other chemicals such as turpentine and ammonia to produce explosive compounds such as chloramine.

Chlorine overexposure can cause a variety of health issues such as blurred vision, coughing, troubled breathing, nausea, vomiting, watery eyes, wheezing, and skin blisters should it encounter the skin. It can induce toxic pneumonitis and severe pulmonary oedema if exposure is between 40 and 60 ppm, and it can be deadly within 30 min of exposure if exposure is above 400 ppm [13]. The immediately dangerous to life and health (IDLH) concentration of chlorine is 10 ppm, while the occupational exposure limit (OEL) is 0.5 ppm. It is not just chlorine gas that can cause respiratory toxicity; other chlorine-containing compounds such as hypochlorous acid, chlorine dioxide, and chloramine can all cause the same symptoms and signs as chlorine gas does [14]. Repeated exposure to chlorine in swimming pools may also be a substantial risk factor for swimmers to develop asthma. Therefore, it is critical to develop suitable chlorine sensors that are both extremely sensitive and selective for use in real-time monitoring.

**Figure 1:**
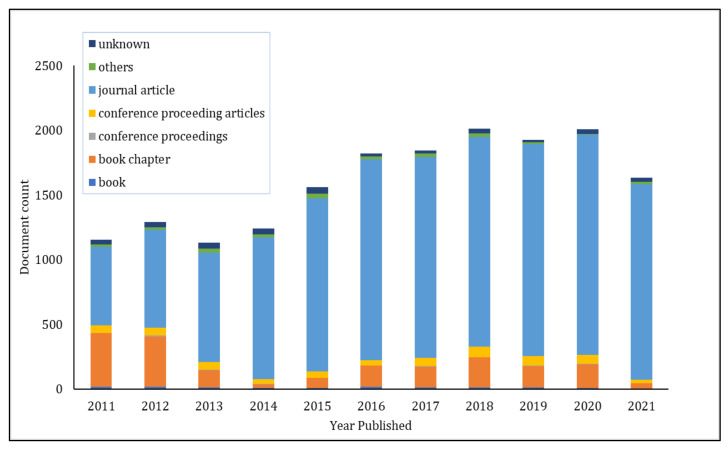
Bar chart of published manuscripts focusing on metal-oxide-based gas sensors.

Semiconductor metal oxide sensors such as tin oxide (SnO_2_), zinc oxide (ZnO_2_), and tungsten oxide (WO_3_) are frequently used as gas sensors [15,16,17,18,19,20]; nevertheless, they require significant power because of their need to operate at high elevated temperatures and also have general selectivity issues. Hence, there is a continuing need for the development of novel gas-sensor material. Apart from magnetic and electrical applications, ferrites are studied for gas- and humidity-sensing applications [21,22,23,24]. Nickel ferrite nanoparticles are useful gas sensors for different gases, such as carbon monoxide (CO), hydrogen disulphide (H_2_S), and chlorine (Cl_2_), and for liquified petroleum gas (LPG) [25,26,27]. However, nickel ferrite nanoparticles as gas sensors receive less attention. Nanoparticles have an efficacious grain size that provides a voluminous surface area to promote the adsorption of numerous gases [28]. The use of nanoparticles in thin-film sensors enhances the sensitivity and performance of gas sensors. Thin-film sensors provide certain advantages such as a high surface-area-to-volume ratio, which improves the interaction between gas analyte and sensing material; lower energy input; low power consumption; compactness; and overall cost efficiency. Consequently, the sensitivity and selectivity of gas sensors using this material are enhanced. As shown in Figure 1, a search on lens.org using the keyword ‘metal-oxide-based gas sensor’ revealed that publications focusing on metal oxides as sensing material have increased in recent years. Therefore, research on metal oxides as gas sensors continues to pique the interest of scientists. 

**Figure 2:**
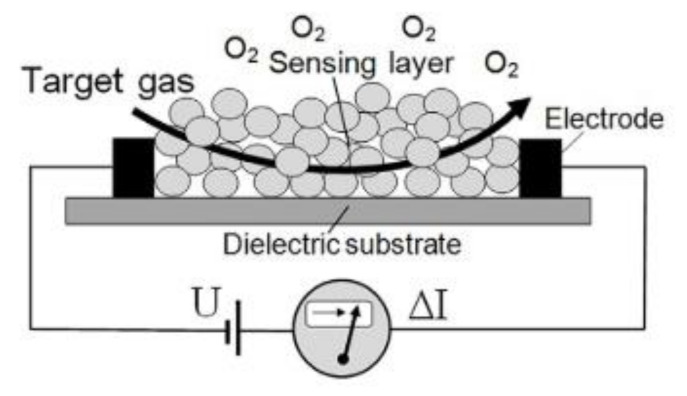
Schematic diagram of metal oxide sensing mechanism [30].

Metal oxide sensing mechanisms are mostly dependent on charge transfer mechanisms, which include oxygen and target analytes (see Figure 2). When the sensing material is operated at temperatures above 150 °C, oxygen molecules from the air chemisorb onto the sensing material’s surface, thus capturing electrons and transforming them into O_2_^−^, O^−^, or O^2−^. When the sensing material is operated at room temperature, the opposite trend occurs. As an electrical charge carrier, the majority carrier is a hole, and the target analyte chemisorbs onto the sensing material’s surface, reducing the concentration of holes, thereby increasing the sensing material’s resistivity [29]. Available papers that report on the efficient gas sensing performance of nickel ferrite nanoparticles are listed in Table 1.

On the other hand, nickel ferrite has the major drawback of tending to agglomerate as a result of its magnetic characteristics, which then impacts its physical characteristics. This necessitates further treatment of ferrite nanoparticles in order to increase their dispersibility. Virlan et al. (2017) demonstrated that heat treatments improved the sensibility of their sensing material. The morphology of mixed Co−Ni ferrites is affected by calcination temperatures, but it has no effect on their magnetic characteristics. The porosity and specific surface area of the material were enhanced, thus improving their sensibility and specificity as a sensor [37]. Ferrite nanoparticles with cellulose nanocrystals (CNC) were modified by Lizundia et al. (2020). They successful fabricated a well-dispersed hybrid material with both magnetic and conductive properties using CNC processes that kept the material homogeneously distributed at the molecular level [38]. Gupta et al. (2017) revealed that the catalytic activity of nanocellulose-nickel ferrite nanocomposites was enhanced due to stabilisation and increased the dispersibility of NiFe_2_O_4_ nanoparticles in a cellulose matrix [39]. Nanocellulose, as an eco-friendly material, can be incorporated with ferrite nanoparticles, thus obtaining organic–inorganic hybrid composites with upgraded functional characteristics. This is due to the presence of hydroxyl groups on the surface of nanocellulose and the interaction of van der Waals forces [40,41] contributing to the attachment of inorganic particles onto the nanocellulose. Furthermore, nanocellulose has a high surface area, a unique morphology, strong mechanical characteristics, and nanoscale dimensions [42,43], thus rendering it an ideal template for stabilising bare ferrite nanoparticles. 

In view of these developments, we attempted to synthesise nickel ferrite loaded onto nanocellulose using an in-situ preparation technique. The nickel ferrite was loaded using different amounts to optimise their sensing characteristics. The structure was examined using XRD analysis, and its elemental composition was evaluated using EDX analysis. Sensing measurements towards chlorine gas were carried out using a fabricated thin-film sensor in a customised test chamber in ambient conditions. The gas concentration, sensitivity, repeatability, and reusability of the sensing material were explored. The combination of structural stability, high surface area, and nickel ferrite and nanocellulose being a well-dispersed composite is an excellent sensing material with vast potential applicability in numerous fields such as biomedicine, wastewater treatment, industrial applications, and for household monitoring.

## 2. Materials and Methods

### 2.1. Materials

Nickel ferrite (NiFe_2_O_4_) was purchased from Alfa (New York, NY, USA). Nanocellulose/cellulose nanofibrilled in water suspension (2% *w*/*v*) was obtained from Zoepnano Sdn. Bhd (Selangor, Malaysia). Liquor ammonia, ethanol, and silver conductive paste were acquired from Merck (Kenilworth, NJ, USA) and used without any pretreatment. A gold ribbon with a thickness of 0.01 mm was purchased from Coining, Ametek (Montvale, NJ, USA).

### 2.2. Nanocomposite Preparation 

**Figure 3:**
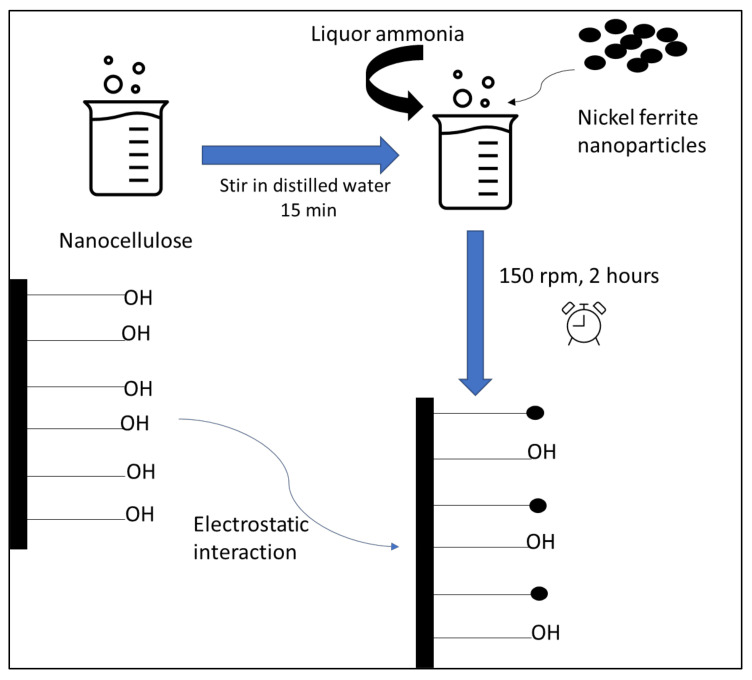
Schematic diagram of preparation nanocomposite.

Nanocellulose was suspended in distilled water and sonicated for 15 min to break up any fibre agglomerations. Then, 0.1 g of nickel ferrite was dissolved in 10 mL distilled water. Drop by drop, the prepared nickel ferrite salt solution was added to the 0.1 g nanocellulose suspension. By adding liquor ammonia, the pH of the solution was adjusted to 7.5, and stirring continued for 2 h. After the reaction had been completed, the nanocellulose–nickel ferrite solution was filtered through a 0.45 µm thick cellulose nitrate membrane filter and rinsed multiple times with distilled water. The end product was dried for 24 h in a vacuum oven at 70 °C. To evaluate the effects of loading different amounts of nickel ferrite towards sensing characteristics, the concentrations of the nickel ferrite solution were varied: 0.1, 0.2, 0.3, 0.4, and 0.5 g. A schematic diagram of the preparation of the nanocomposite is shown in Figure 3.

### 2.3. Characterisation of Hybrid Nanocomposites

#### 2.3.1. X-ray Diffraction Analysis

X-Ray diffraction analysis (XRD) is a non-destructive method used to identify the crystalline structure of samples. XRD patterns of the nanocellulose, nickel ferrite, and nanocellulose-nickel ferrite nanocomposites were recorded using a Smart APEX II Bruker AXS GmbH (Karlsruhe, Germany), and Bragg’s scanning angle varied from 10° to 90°. Samples were examined at ambient temperature with Cu–Kα radiation (wavelength 1.54 Å). Data analysis began with the subtraction of a blank run to remove the environmental background before sample crystallinity was analysed.

#### 2.3.2. Energy-Dispersive X-ray Analysis

Energy-dispersive X-ray (EDX) is a technique used to examine the elemental composition of a sample. EDX analysis was integrated in a Zeiss Gemini500 SEM instrument (Jena, Germany). Elemental composition presence in the nanocomposites is presented in the form of percentages. Nickel and iron elements appeared as indications of the attached nickel ferrite.

### 2.4. Sensing Measurements

#### 2.4.1. Preparation of Thin-Film Sensing Materials

Ethanol was used to clean a prepatterned gold (Au) interdigitated transducer (IDT) with a 50 µm line/space. The IDT was then annealed in an oven for 15 min at 50 °C to evaporate any remaining solvent. Using the silver conductive paste, a gold ribbon (99.9% purity) was placed onto the gold (Au) pads and allowed to dry and harden under a heated environment of 60 °C for 15 min. Approximately 2.5 µL of the hybrid nanocomposite solution was then drop-cast onto the IDT using an Eppendorf micropipette. The prepared IDT was then dried at room temperature for 24 h. The same technique was used to prepare the pristine nanocellulose and bare nickel ferrite IDTs as controls in the experiment. 

#### 2.4.2. Detection of Chlorine Gas

**Figure 4:**
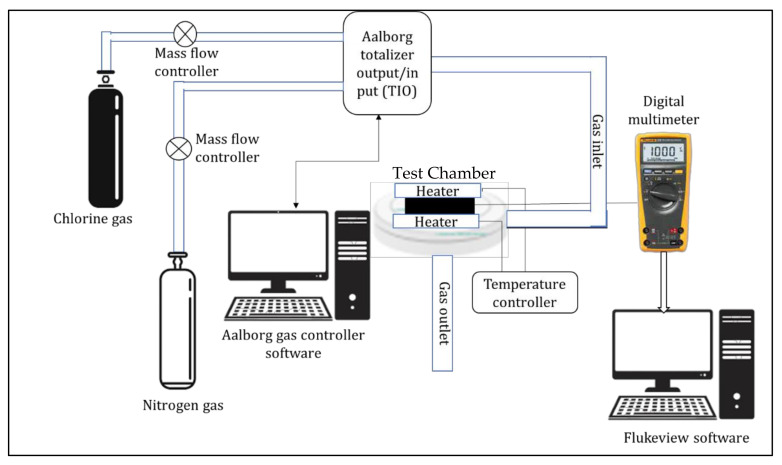
Sensor test setup.

As is shown in Figure 4, IDTs loaded with the nanocomposites were installed in a designed gas test chamber and attached to a digital multimeter and temperature controller. The digital multimeter was connected to a computer running FlukeView form software to record the samples’ electrical resistance changes in real time. The gas test chamber was integrated into a gas calibration system that included a computer-controlled mass flow controller that regulated gas flow at 200 standard cubic centimetres per minute (sccm). The concentration of chlorine gas was changed by regulating the dilution of 1% chlorine gas with an inert carrier gas, in this case, nitrogen. The resistance was then continuously measured while the sample was subjected to various concentrations of chlorine gas. The dynamic response obtained from each of the sample IDTs was observed as a change in resistance when the test chamber was alternately filled and purged with chlorine and nitrogen gas. Measurements were taken at ambient temperature and in a constant humidified atmosphere (55%). Equation (1) was used to calculate the sensitivity of the sensing material:

(1) $$S=\frac{\Delta R}{R_{o}}=\frac{R_{g}-R_{o}}{R_{o}}\times 100\%$$

where S denotes the sensitivity of the sensing material, R_g_ denotes the resistance of the sample upon exposure to chlorine gas, and R_o_ denotes resistance in the sample upon exposure to nitrogen gas.

The repeatability study was conducted using the same material at optimal concentrations and injected with 1% chlorine. The reusability study was conducted using the same material at optimal concentrations, but in five different days. The change in resistance was measured using the same calculation as above.

## 3. Results and Discussion

### 3.1. Characterisation

**Figure 5:**
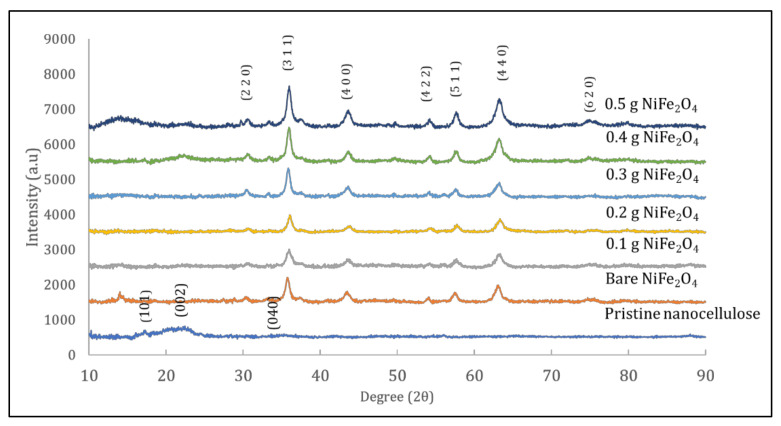
XRD pattern of pristine nanocellulose, bare NiFe_2_O_4_, and hybrid nanocomposites.

Figure 5 displays the X-ray diffraction patterns of nanocellulose, nickel ferrite, and the hybrid nanocomposites comprising five different NC weight loading levels (0.1, 0.2, 0.3, 0.4, and 0.5 g). All peaks were prominent, and matched well with JCPDS card no. 00-050-2241 and JCPDS card no. 10-0325, which indicated the crystalline structure of the pristine nanocellulose and the spinel cubic structure of pure nickel ferrite nanoparticles. The XRD diffraction pattern showed peaks around 2θ = 17.69° (101), 22.08° (002) and 34.51° (040), which indicated a typical cellulose structure [39]. The matched scan data of NiFe_2_O_4_ showed the formation of crystalline nickel ferrite nanoparticles, as indicated in JCPDS card no. 00-003-0875. Obtained diffraction patterns were 31.01°, 35.99°, 43.72°, 54.49°, 57.89°, 63.27°, and 75.05°, which were assigned to the reflection of planes (220), (311), (400), (422), (511), (440), and (620), respectively [28]. We did not observe any extra peaks that could indicate the presence of any impurities in the prepared sample, thereby confirming the purity of the sample. In the XRD pattern of the nanocomposites, besides typical peaks of nanocellulose at 2θ = 22.73° (002), there were some other diffraction peaks that matched well with the standard pattern of NiFe_2_O_4_. As the concentration of nickel ferrite increased, peaks corresponding to NiFe_2_O_4_ became prominent and intense, while peaks corresponding to nanocellulose decreased in intensity, as is shown in Figure 5. Crystallite size was calculated from the line broadening of the most intense (0 0 2) and (3 1 1) peaks of nanocellulose and nickel ferrite using the Scherrer equation [44]. In X-ray diffraction and crystallography, the Scherrer equation is a formula that relates the size of submicrometric crystallites in a solid to the broadening of a peak in a diffraction pattern. Equation (2) shows the modified Scherrer equation formula [45]. Bragg’s equation was used to calculate lattice parameters. The values of the lattice parameter were in the range of 8.1810–8.2690. The values of crystallite size and lattice parameters of nanocellulose and its composites with nickel ferrite are shown in Table 2.

(2) $$D=\frac{\lambda }{3\beta \cos\theta }$$

where 1/3 is the correction value of shape factor K in the Scherrer equation; λ is the X-ray radiation wavelength; β and θ are the full-width half maximum and the half of the diffraction angle (2θ), respectively, at the crystal planes detected in the XRD spectrum.

**Figure 6:**
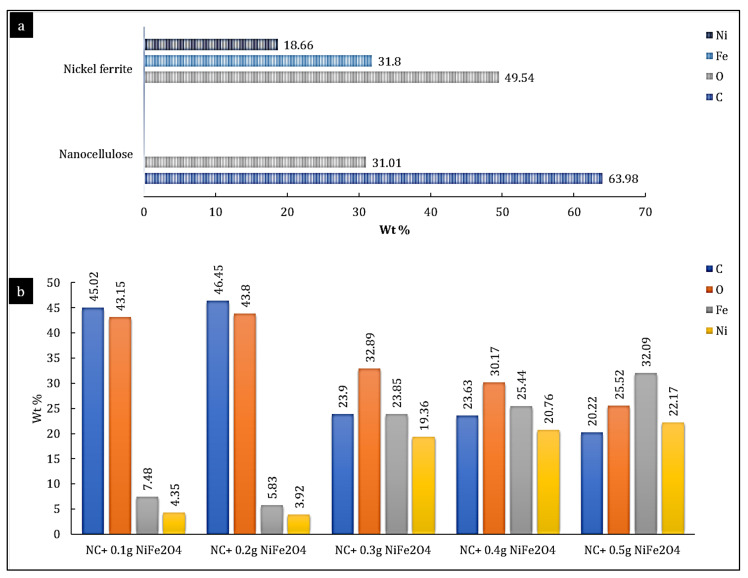
Elemental composition of (**a**) nanocellulose and nickel ferrite (**b**) hybrid nanocomposites.

Samples were then examined using energy-dispersive X-ray spectroscopy analysis (EDX) to evaluate the ratio between divalent cations contained in the nanoparticles to further validate the occurrence of mixed ferrites. The elemental composition of pristine nanocellulose and bare nickel ferrite is shown in Figure 6a. Findings revealed that nanocellulose contained carbon, oxygen, hydrogen, and a small number of impurities (not shown in the graph). Impurities were most likely due to sulphuric acid from the production process [46]. Figure 6a also confirms the purity of nickel ferrite through the presence of Ni, Fe, and O elements, which agreed well with the theoretical values (Fe: 48.97%, Ni: 24.42%, and O: 29.61%) [47]. The hybrid nanocomposites possessed material compositions that were similar to the desired ratios, as shown in Figure 6b. Minor differences could be attributed to technical errors, but overall results indicated the production of tertiary ferrites with well-defined composition. EDX results also corresponded well with lattice parameters estimated from XRD data, thus doubly validating the composition of the nanocomposites [48]. Ferrite ions were successfully diffused and adsorbed onto the nanocellulose surface when the nickel ferrite was submerged in a mixture of nanocellulose solutions via electrostatic interactions. Upon the addition of liquor ammonia, the basic hydrolysis of ferrite ions occurred, inducing a nickel ferrite nanoparticle colloidal solution to form, and the solution darkened as a result of the production of the nickel ferrite nanoparticles. The suspension was well-dispersed and stable because of the strong interactions between nickel ferrite and nanocellulose [39].

### 3.2. Sensing Characteristics

#### 3.2.1. Pristine Nanocellulose and Nickel Ferrite

**Figure 7:**
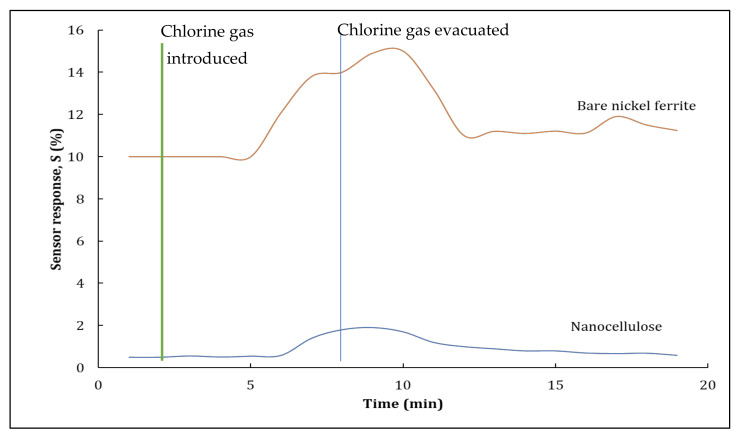
Sensor response of pristine nanocellulose and bare nickel ferrite.

Sensing characteristics of the hybrid nanocomposites as a thin-film sensor were studied by exposing them to chlorine gas at ambient temperature. In the control experiment, a sample without any hybrid nanocomposites was placed in the customised test chamber to evaluate any differences in the responses of a pristine sample and the hybrid nanocomposites. Responses for pure nanocellulose and nickel ferrite nanoparticles are both shown in Figure 7. The thin-film sensor’s resistance was initially examined in a nitrogen atmosphere before the introduction of any other gas into the customised test chamber. The chamber was then filled with 1% chlorine gas. The response of the nanocellulose-based thin film sensor was significantly lower due to the weak conductivity of the nanocellulose. On the other hand, the nickel ferrite nanoparticle thin-film sensor sample exhibited increased resistance as its sensor response. As nickel ferrite is highly conductive, it can promptly respond to the presence of target analytes. After the chlorine gas in the test chamber had reached a steady state, it was purged from the chamber by introducing nitrogen gas. The thin-film sensor’s baseline recovered within several minutes after the evacuation of chlorine gas from the test chamber, indicating that the nickel ferrite nanoparticles displayed a recovery phase within a short span of time. After these control experiments had been completed, hybrid nanocomposites were placed in the customised test chamber to analyse their sensing capabilities.

#### 3.2.2. Nickel ferrite–nanocellulose nanocomposites

**Figure 8:**
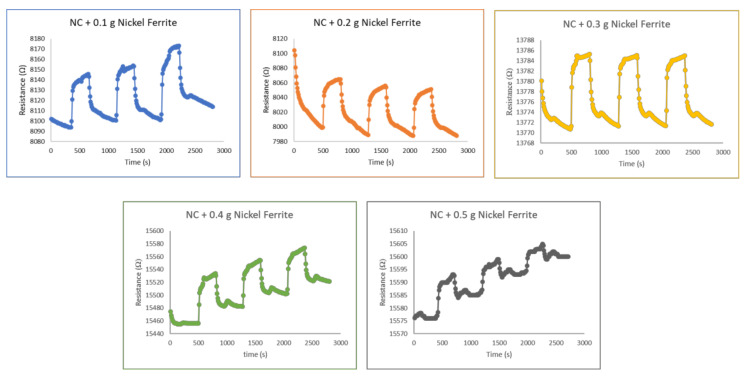
Response of nickel ferrite–nanocellulose nanocomposites to 1% chlorine gas.

The responses of the different quantities of nickel ferrite samples deposited onto the nanocellulose are represented in Figure 8. The magnetic characteristics of ferrite particles were stabilised by using nanocellulose as a substrate. Upon the introduction of chlorine gas into the test chamber, all samples demonstrated a good response. When the sensing materials were exposed to chlorine gas, the chlorine attracted electrons from the sensing materials, thus increasing their electrical resistance. When the chlorine gas was withdrawn, the reaction reversed. Trapped electrons returned to the sensing material, thus increasing the density of electrons in the material, and this lead to a decrease in electrical resistance. The basic mechanism of semiconductor metal oxides is based on charge transfer. The behaviour in this experiment indicated that the sample was a *p*-type semiconductor, this being a majority carrier of holes. In a *p*-type semiconductor, the tested gas draws electrons from the sensing material and thus increases the concentration of holes, resulting in an increase in resistance [49]. 

Since Cl_2_ gas detection is a surface phenomenon, the number of accessible active sites on the surface of a sensor is critical in determining the effectiveness of the sensing material. However, this experiment revealed that the samples were unable to immediately recover to their baseline resistance value. This was very likely due to the tested gas being chemisorbed onto the surface of the sensing medium. The adsorption of gas analytes onto sensing materials can be classified into two types: physisorption and chemisorption. Physisorption occurs when the molecular interaction occurring is mostly driven by van der Waals forces, whereas chemisorption happens when valence forces such as those found in the formation of chemical pollutants are involved. Both adsorption processes can occur on the same surface and are influenced by any increases in surface area [50]. The interaction between gas analyte and sensing material in our situation was most likely chemisorption, which then resulted in the samples having difficulty in recovering their baseline resistance values. This could be remediated through the use of heat treatment or UV illumination to facilitate electron release from the sensing material. The baseline could not be recovered in this experiment, probably because active sites on the sensing material continued to be saturated with the gas analyte and were difficult to remove. In a similar experiment, Sharma et al. (2018) described a similar phenomenon in which a chlorine sensor based on carbon nanotubes modified with hexadecafluorinated copper phthalocyanine failed to regain its baseline resistance, even after a long period of time. Nonetheless, they found that adding heat to the sensing materials greatly enhanced the material’s recovery properties [51].

#### 3.2.3. Sensitivity Study of Hybrid Nanocomposites

**Figure 9:**
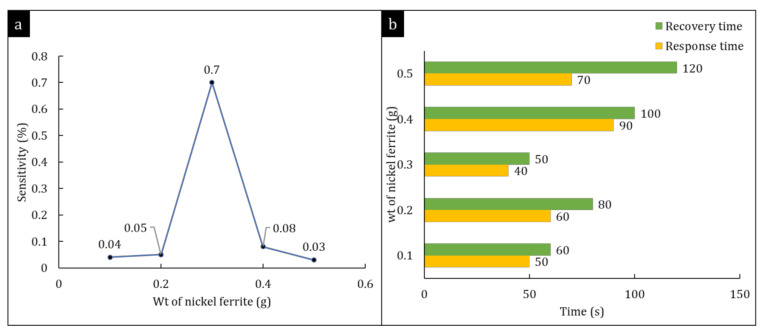
(**a**) Sensitivity of different amounts of nickel ferrite towards chlorine gas. (**b**) Response and recovery time of different hybrid nanocomposites.

Figure 9a illustrates the sensitivity of a hybrid nanocomposite after being exposed to 1% chlorine gas. With respect to the nickel ferrite, the use of different amounts, namely, 0.1, 0.2, 0.3, 0.4, and 0.5 g produced sensitivities of 0.04%, 0.05%, 0.7%, 0.08%, and 0.05%, respectively. Nickel ferrite has outstanding sensing properties, such as great conductivity, high surface area, and the presence of two different cation sites. Combining cations in the ferrite particles opens up the possibility of designing promising sensing materials with improved sensitivity, selectivity, responsiveness, and long-term stability [52]. Furthermore, nickel ferrite has a lower reaction barrier compared to that of other metals (see Table 3), which renders them highly effective at dissociating chlorine molecules, resulting in a rapid response to the presence of chlorine gas. Increasing the quantity of nickel ferrite promotes an increased number of active sites for Cl_2_ gas adsorption on the surface of the sensing material. The deposition of nickel ferrite onto nanocellulose is expected to enhance their dispersibility in the material. Thus, well-dispersed ferrite nanoparticles on the surface of nanocellulose would enable extensive contact with gas analytes, thereby increasing the sensing material’s sensitivity. However, once the concentration of nickel ferrite exceeded 0.3 g, sensitivity was reduced. This was likely because nickel ferrite tends to agglomerate on surfaces. This agglomeration limits interactions between gas analytes and the sensing material, hence resulting in reduced sensitivity. Rao et al. (2016) reported that nickel ferrite doped with palladium in a thin-film Cl_2_ sensor had a sensitivity of 6.9 at a working temperature of 325 °C [31]. Zhang et al. (2019) utilised a three-dimensional open porous tin dioxide (SnO_2_) chlorine sensor with a high sensitivity of 792.85 to 5 ppm Cl_2_ at 160 °C [53]. Even though the sensitivity of 0.3 g nickel ferrite coated onto nanocellulose had a significantly lower value (0.7), the needed operating temperature was room temperature, which indicated that minimal maintenance would be needed, and there would be lower manufacturing costs in a room-temperature sensor device for chlorine monitoring applications.

In effective gas sensors, response and recovery characteristics are important criteria to be considered. When a sensor is exposed to gas, its response time is defined as the time taken for the sensor to achieve 90% of the entire current/resistance/voltage change, and recovery time is the time that it takes to remove the gas from the sensor. In our study, 0.3 g of nickel ferrite demonstrated promising response and recovery times of 40 and 50 s, respectively. Hybrid nanocomposite samples showed varying response times, from 50 to 90 s, and recovery times from 60 to 120 s (Figure 9b). The 0.3 g nickel ferrite thin film had the fastest reaction time and recovery time, suggesting that it would be a better sensor. Bedi et al. (2010) reported that their copper (II) 1,4,8,11,15,18,22,25-octabutoxy-29H,31H- phthalocyanine thin film as a Cl_2_ gas sensor that could operate at room temperature with high sensitivity (185%), but it had a response time of 9.5 min. The sensor also needed to be heated to 195 °C in order to achieve recovery [54]. High sensitivity (50%) could also be obtained using NiZnFe_2_O_4_ to detect 1000 ppm Cl_2_ at room temperature, but response and recovery times were much longer (10 and 13 min, respectively) [44] than those found in this study.

**Table 3:** Metal reaction barrier.

Metal Oxides	Reaction Barrier	Ref.
Metal atoms (X = Cu, Zn, Cd, Ga, Al, Au, or Hg) with YH_4_ molecules (Y = C, Si, or Ge)	85–109.8	[55]
Ga-21.5In-10Sn/Cu	98–106.7	[56]
MgCl_2_	290.2	[57]
NiFe_2_O_4_	~7	[58]

**Figure 10:**
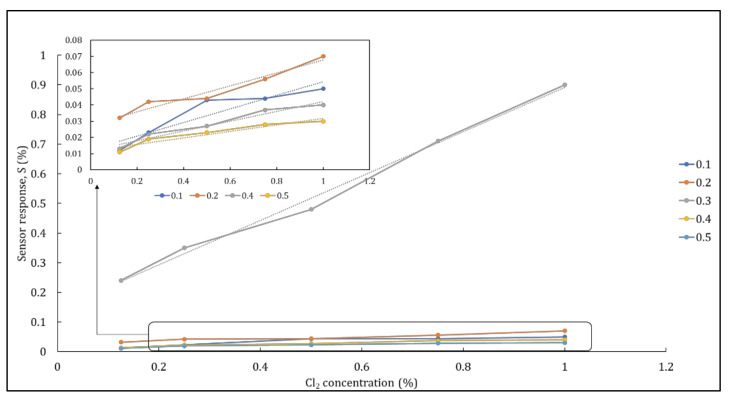
Sensor response of nickel ferrite–nanocellulose nanocomposite towards different chlorine concentrations.

Figure 10 depicts the different hybrid nanocomposite responses as a function of gas concentration. The sensitivity of these different hybrid nanocomposites was compared at 25 °C. The sensor response of the nickel ferrite–nanocellulose nanocomposites was about 0.24%, 0.35%, 0.48%, 0.71%, and 0.90% for 0.3 g nickel ferrite loaded nanocomposite when exposed to Cl_2_ gas concentrations of 0.125%, 0.25%, 0.5%, 0.75%, and 1.0%, respectively. The sensor response of the other hybrid nanocomposites, their fitting function, and the correlation coefficients of the fitted data are shown in Table 4. These observations revealed that there was a comparative increase in sensor response with increasing gas concentrations. The gas detection mechanism of a metal oxide semiconductor to a reducing gas (Cl_2_) is described as follows. When the sensing material is exposed to a targeted gas, resistance changes depend on the interaction between gas analyte and sensing material. The adsorption of the gas analyte on the sensing material can influence response characteristics. A better response might be predicted when a substantial amount of gas analyte is adsorbed, increasing the sensitivity of the sensing material [59]. Hence, increases in sensor response as gas concentration increases are attributable to the presence of active sites on the sensing material. When larger amounts of gas analytes react at the active sites, this improves the adsorption of the gas, thus increasing the sensor response. 

#### 3.2.4. Repeatability and Reusability of Hybrid Nanocomposites

**Figure 11:**
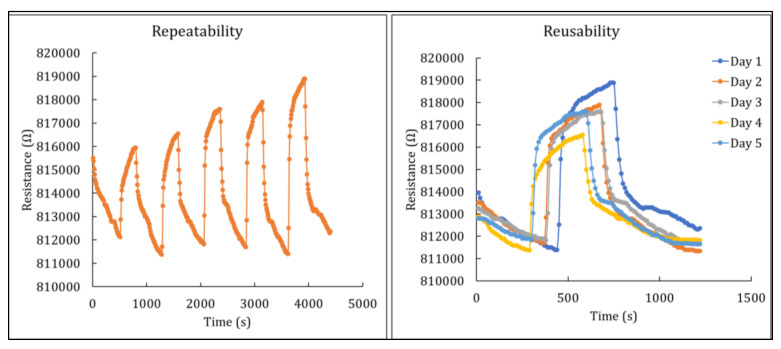
Repeatability and reusability characteristics of nickel ferrite–nanocellulose nanocomposite.

Figure 11 exhibits the repeatability and reusability characteristics of the sensing material, which were examined using 0.3 g of nickel ferrite deposited onto the nanocellulose. The sensing material was exposed to 1.0% Cl_2_ gas for five times in order to examine its repeatability, and the same sensing material was then exposed to a fixed concentration of Cl_2_ gas over five consecutive days to examine its reusability. The sensing material was flushed with nitrogen gas to recover its baseline resistance value without any exposure to any form of heat treatment. The sensing material demonstrated good repeatability and reusability through not exhibiting any significant decrease in sensing performance throughout the tests. However, possibly due to a small amount of the gas analyte thoroughly penetrating the surface of the ferrite nanoparticles, there was a minor difference in the resistance value during the recovery phase, which may prove to be negligible. This finding indicates that the nickel ferrite–nanocellulose nanocomposite has considerable potential as a sensing material for monitoring hazardous gases, with good stability and recovery after exposure.

## 4. Conclusions

Nickel ferrite–nanocellulose nanocomposites could be synthesised using a simple in situ preparation method. In this study of the sensing properties of nickel ferrite, five different weight ratios of nickel ferrite were deposited onto nanocellulose. Nanocomposites exhibited a consistent crystallite size in the nanoscale according to performed XRD measurements. When compared to other weight ratios, 0.3 g nickel ferrite demonstrated improved responsiveness and a shorter recovery time at room temperature. The reaction site in nickel ferrite provided two different cation sites, which promoted high sensitivity in the sensing material. Furthermore, nickel ferrite has a lower reaction barrier compared to other metals, rendering it highly effective at dissociating chlorine molecules, thereby demonstrating a rapid response to chlorine gas. As such, it has promising future applications for the detection of low concentrations of chlorine gas at room temperature. We aim to continue improving the sensitivity and selectivity of this sensing material in our laboratory. The selectivity of multiple gases using the nanocomposite material can be examined using principal component analysis (PCA). Additionally, the adsorption of various gas analytes on the nanocomposites can be studied by first-principle calculations using density functional theory (DFT). Binding energy, charge transfer, and morphology distance are generally investigated. Hence, electron density analysis could identified that the induction of electron transfer from C atoms in the nanocomposite towards gas analytes leads to the hole (or *p*-type) doping of semiconductor nanocomposites.

## Figures and Tables

**Table 1:** Reports of efficient gas-sensing performance of nickel ferrite nanoparticles.

Sensing Material	Target Gas	Operating T (°C)	Concentration of Gas (ppm)	R_T_ and R_C_	S (%)	Ref.
Pd-doped NiFe_2_O_4_	Cl_2_	325	15	2 s and 6 s	6.9	[31]
Co-doped NiFe_2_O_4_	LPG	250	5000	25 s and 240 s	86	[32]
Li-doped NiFe_2_O_4_	H_2_	200	100	Not mentioned	1.09	[33]
Nb_2_O_5_ mixed with NiFe_2_O_4_	Humidity	25	11–97 %	20 s and 30 s	1190	[34]
Ru−NiFe_2_O_4_	H_2_	100	50	Not mentioned	1.36	[35]
NiFe_2_O_4_	Triethylamine	190	50	6 s and 585 s	18.9	[36]

T: temperature; R_T_: response time; R_C_: recovery time; S: sensitivity.

**Table 2:** Lattice parameters and average crystallite size of nickel ferrite nanocellulose.

Sample	Lattice Parameters (Å)	Average Crystallite Size, D (nm)	
		Nanocellulose	NiFe_2_O_4_
Pristine NC	−	1.40	−
Bare NiFe_2_O_4_	8.2690	−	0.83
NC + 0.1 g NiFe_2_O_4_	8.1810	1.34	0.96
NC + 0.2 g NiFe_2_O_4_	8.2010	1.28	0.97
NC + 0.3 g NiFe_2_O_4_	8.2125	1.22	0.98
NC + 0.4 g NiFe_2_O_4_	8.2183	1.33	0.83
NC + 0.5 g NiFe_2_O_4_	8.2203	1.25	0.93

**Table 4:** Sensor response, fitting function, and correlation coefficients (R^2^) of nickel ferrite–nanocellulose nanocomposites.

Hybrid Nanocomposite	Concentration (%)					Fitting Function	R^2^
	0.125	0.25	0.5	0.75	1.0		
NC + 0.1 g NiFe_2_O_4_	0.012	0.023	0.043	0.044	0.05	y = 0.0419x + 0.0124	0.8622
NC + 0.2 g NiFe_2_O_4_	0.032	0.042	0.044	0.056	0.07	y = 0.0398x + 0.0279	0.9522
NC + 0.3 g NiFe_2_O_4_	0.240	0.350	0.480	0.710	0.90	y = 0.7473x + 0.1437	0.9934
NC + 0.4 g NiFe_2_O_4_	0.013	0.022	0.027	0.037	0.04	y = 0.0300x + 0.012	0.9506
NC + 0.5 g NiFe_2_O_4_	0.011	0.019	0.023	0.028	0.03	y = 0.0202x + 0.0116	0.9056

## Data Availability

The data presented in this study are available on request from the corresponding author.
